# Glimepiride-induced cholestasis in a man with diabetes mellitus: a case report

**DOI:** 10.4076/1752-1947-3-9257

**Published:** 2009-09-15

**Authors:** Hesham Omar, Jaya Kolla, Devanand Mangar, Enrico Camporesi

**Affiliations:** 1 Department of Cardiovascular Medicine, Cairo University Hospital, Cairo, Egypt; 2 Tampa General Hospital, Tampa, FL, USA; 3 Florida Gulf to Bay Anesthesiology, Tampa, FL, USA; 4 University of South Florida, Tampa, FL, USA

## Abstract

**Introduction:**

There has been a progressive increase in the incidence of type II diabetes mellitus cases over the past decade. The availability of a wide variety of oral hypoglycemics has given rise to a number of adverse effects. In this case report, we describe the case of a patient recently diagnosed with type II diabetes. He was receiving treatment with glimepiride and experienced rapid onset of cholestatic liver injury as a side effect, which reversed upon cessation of therapy.

**Case presentation:**

We present the case of a 58-year-old Egyptian man with a recent diagnosis of type II diabetes mellitus. He presented with a clinical picture of progressive jaundice three days before admission. Laboratory investigations revealed elevated bilirubin, alkaline phosphatase and gamma glutamyl transferase levels. Ultrasonography revealed intrahepatic biliary duct dilatation and two small lymph nodes in the porta hepatis; there was, however, no extrahepatic biliary duct dilatation or stones in the gall bladder. Abdominal computed tomography excluded pancreatic or hepatic focal lesions, but the tumor marker CA19-9 was elevated. The progressive improvement in the patient's symptomatology and laboratory investigations after admission argued against malignancy. A thorough and detailed history revealed that the patient had started on a new medication, glimepiride, five months earlier for the treatment of diabetes mellitus and an improvement was noted after discontinuation of this oral hypoglycemic and the introduction of insulin therapy to control his blood sugar.

**Conclusion:**

Drugs are an important, often unrecognized, cause of acute cholestasis. Among the rare causes responsible for this complication is the sulfonylurea glimepiride. A thorough drug history is therefore helpful in any case of unexplained cholestasis.

## Introduction

The incidence of diabetes mellitus has been steadily rising as a consequence of sedentary lifestyles and poor dietary habits. A wide variety of oral medications has recently become available for the management of diabetes mellitus. Sulfonylureas are one of the drug classes that act by blocking the pancreatic β-cell potassium channel causing depolarization and subsequent insulin release. Sulfonylureas are widely prescribed drugs and are an infrequent cause of cholestatic liver injury. However, in this case report, we describe a more unusual presentation caused by a second generation sulfonylurea, glimepiride.

## Case presentation

The patient was a 58-year-old Egyptian man with a 40-year history of cigarette smoking and a medical history of chronic cholecystitis. He had been recently diagnosed with diabetes mellitus. He was started on a treatment regimen of glimepiride 3 mg daily, as well as undertaking dietary and lifestyle modifications, resulting in adequate control of his blood sugar levels.

Five months into therapy, the patient started noticing fever, rigors and persistent vomiting and attributed these symptoms to an active flare-up of chronic cholecystitis. Antibiotic treatment was initiated with no improvement in symptomatology. Two days later, the patient started noticing yellowish discoloration of his eyes followed by darkening of his urine and lighter colored stools. The patient sought medical advice and was admitted to the hospital for further work-up.

On admission, initial laboratory investigations revealed a total serum bilirubin of 5 mg/dl, mainly of the direct fraction. The serum alkaline phosphatase level was 300 U/l, the serum glutamic pyruvic transaminase (SGPT) level was 90 U/ml and the serum glutamic oxaloacetic transaminase (SGOT) level was 70 U/ml. SGPT levels were always higher than SGOT. The patient's uncontrolled blood glucose required the replacement of the oral hypoglycemic with insulin for tight glucose control.

A preliminary diagnosis of cholestatic viral hepatitis vs. calcular obstructive jaundice (due to his long history of chronic cholecystitis) was presumed. Viral hepatitis serology was performed and was negative for hepatitis A virus (HAV), hepatitis B virus (HBV) and hepatitis C virus (HCV). Further ultrasonographic evaluation showed evidence of dilated intrahepatic biliary ducts, a contracted gall bladder with thickened walls and two small lymph nodes present in the porta hepatis. However, there was no evidence of gall stones or dilatation of the extrahepatic biliary ducts. Magnetic resonance cholangiopancreatography (MRCP) was performed and revealed no evidence of stones in the common bile duct and no extrahepatic biliary duct dilatation.

A gastrointestinal carcinoma, a pancreatic carcinoma or a cholangiocarcinoma were suspected, with possible lymphadenopathy in the porta hepatis causing obstructive jaundice. A computed tomography (CT) scan of the abdomen confirmed the presence of two lymph nodes in the porta hepatis and the tumor marker CA19-9 reached 300U/ml, increasing the possibility of a malignancy.

During our patient's stay in hospital, there was progressive improvement in his symptomatology as well as the laboratory investigations, evident by progressive reductions in the serum bilirubin, alkaline phosphatase, serum gamma glutamyl transferase (GGT) and tumor marker CA19-9. This improvement in symptomatology, and the laboratory investigations and the radiologic evidence argued against malignancy as an etiology for the described presentation, which would have otherwise caused progressive worsening of the patient's condition. This raised the possibility that there might be another underlying cause of cholestasis. After a detailed and thorough review of the patient's history, the recent addition of glimepiride for treatment of his diabetes mellitus was noticed. We suspected that this newly introduced medication was responsible for the patient's presentation. The progressive improvement in the patient's condition was attributed to the discontinuation of his oral hypoglycemic medication after admission and the start of insulin therapy for the control of his blood glucose.

Five days after admission, the patient's general condition dramatically improved and he was subsequently discharged from hospital with a recommendation of regular follow-up of serum bilirubin and tumor marker CA19-9. The patient's liver profile returned to normal within two months. The initial ultrasound findings of intrahepatic bile duct dilatation and lymph nodes enlargement, as well as the elevated CA 19-9 level had resolved and normalized, this excluded malignancy. Lymph node enlargement can, however, be explained by the presence of associated cholangitis or hepatitis and elevated CA19-9 levels can occur in cholestasis, especially if levels are not so high [1]. One month after discharge, the patient's laboratory results revealed normalization of serum bilirubin, hepatic transaminases and CA 19-9 levels; however, serum alkaline phosphatase levels took two months to normalize. After he was discharged, the patient was prescribed metformin as a substitute for glimepiride for the control of blood glucose.

## Discussion

Drugs are an important, often unrecognized, cause of acute cholestasis. The clinical presentation and laboratory picture of acute cholestasis resemble that of other hepatobiliary disorders,for example, calcular obstructive jaundice. A detailed and thorough drug history should be taken by the clinician in any patient presenting with acute cholestasis. Discontinuation of the offending agent causes a dramatic improvement in the patient's symptomatology, and prevents adverse outcomes [2].

A wide variety of drugs can potentially cause this disorder including oral contraceptive pills, anabolic steroids, testosterone, chlorpromazine, fibrates and nifedipine. Certain antibiotics such as erythromycin, flucloxacillin, co-amoxicillin, nitrofurantoin and trimethoprim sulfamethoxazole [3] should also be considered. Cholestasis has rarely been encountered in clinical practice in patients with diabetes receiving sulfonylureas, especially glimepiride. There is only one other article in the literature that attributed this complication to glimepiride, however, the diagnosis in that case was confounded by the presence of a stone in the common bile duct [4].

Patients with acute cholestasis usually present with pruritis, jaundice (usually olive green discoloration of the sclera), dark urine and clay colored stools. Laboratory investigations might show evidence of conjugated hyperbilirubinemia, elevated serum GGT and 5' nucleotidase levels. Mild elevation of serum transaminases may also be noticed as a result of the toxic effect of acute bile retention on hepatocytes or concomitant hepatitis.

Drug-induced cholestasis can be divided into acute and chronic forms [5]. The acute form is further subdivided into cholestasis without inflammation ('bland' cholestasis), cholestasis with inflammation, and cholestasis with bile duct injury. The chronic form includes a vanishing bile duct syndrome and a sclerosing cholangitis-like syndrome. Drugs that cause cholestasis with a bile duct injury are usually accompanied by additional clinical features such as fever, rigors, jaundice, and tender hepatomegaly mimicking that seen in acute cholangitis. Drugs that result in a vanishing bile duct syndrome may cause rapidly progressive cholestasis with prolonged periods of jaundice and occasionally cirrhosis and liver cell failure.

Drug-induced acute cholestasis is a diagnosis of exclusion, therefore, hepatobiliary imaging is a significant step in the diagnosis of patients presenting with this disorder. Hepatobiliary imaging is essential to exclude the presence of extrahepatic biliary duct dilatation whether due to pancreatic carcinoma, or a common bile duct stone and to exclude the presence of any hepatic or pancreatic mass lesions.

Management of drug-induced cholestasis is focused mainly on immediate discontinuation of the offending drug if it is recognized and observing the patient for clinical and laboratory improvement. Symptomatic treatment for vomiting, fever and especially pruritis should also commence. Prognosis of drug-induced cholestasis is usually good and recovery is the rule in most cases, however, severe cases may progress to liver cirrhosis and eventually liver failure.

## Conclusion

Drugs are an important, often unrecognized, cause of acute cholestasis. Drug-induced hepatotoxicity has been infrequently reported with the use of sulfonylureas and especially glimepiride. To the best of our knowledge, this is the first report in the literature where the diagnosis was not confounded by any other etiology. Diagnosis should be considered in any patient with type II diabetes mellitus on oral hypoglycemic agents presenting with unexplained cholestatic jaundice. A thorough drug history should be recorded by the practitioner in any case of unexplained cholestasis. Recovery is common in these cases provided that the drug is identified and discontinued.

## Abbreviations

CT: computed tomography; GGT: gamma glutamyl transferase; HAV: hepatitis A virus; HBV: hepatitis B virus; HCV: hepatitis C virus; MRCP: magnetic resonance cholangiopancreatography; SGPT: serum glutamic pyruvic transaminase; SGOT: serum glutamic oxaloacetic transaminase.

## Consent

Written informed consent was obtained from the patient for publication of this case report. A copy of the written consent is available for review by the Editor-in-Chief of this journal.

## Competing interests

The authors declare that they have no competing interests.

## Authors' contributions

HO and JK were responsible for drafting the manuscript and the literature search. HO was responsible for diagnosis of the case. EC and DM both made critical revisions to the manuscript. All authors have read and approved the final manuscript.
